# Enabling Global Clinical Collaborations on Identifiable Patient Data: The Minerva Initiative

**DOI:** 10.3389/fgene.2019.00611

**Published:** 2019-07-29

**Authors:** Christoffer Nellåker, Fowzan S. Alkuraya, Gareth Baynam, Raphael A. Bernier, Francois P.J. Bernier, Vanessa Boulanger, Michael Brudno, Han G. Brunner, Jill Clayton-Smith, Benjamin Cogné, Hugh J.S. Dawkins, Bert B.A. deVries, Sofia Douzgou, Tracy Dudding-Byth, Evan E. Eichler, Michael Ferlaino, Karen Fieggen, Helen V. Firth, David R. FitzPatrick, Dylan Gration, Tudor Groza, Melissa Haendel, Nina Hallowell, Ada Hamosh, Jayne Hehir-Kwa, Marc-Phillip Hitz, Mark Hughes, Usha Kini, Tjitske Kleefstra, R Frank Kooy, Peter Krawitz, Sébastien Küry, Melissa Lees, Gholson J. Lyon, Stanislas Lyonnet, Julien L. Marcadier, Stephen Meyn, Veronika Moslerová, Juan M. Politei, Cathryn C. Poulton, F Lucy Raymond, Margot R.F. Reijnders, Peter N. Robinson, Corrado Romano, Catherine M. Rose, David C.G. Sainsbury, Lyn Schofield, Vernon R. Sutton, Marek Turnovec, Anke Van Dijck, Hilde Van Esch, Andrew O.M. Wilkie, The Minerva Consortium

**Affiliations:** ^1^Nuffield Department of Women’s and Reproductive Health, University of Oxford, Oxford, United Kingdom; ^2^Big Data Institute, University of Oxford, Oxford, United Kingdom; ^3^Institute for Biomedical Engineering, University of Oxford, Oxford, United Kingdom; ^4^Department of Genetics, King Faisal Specialist Hospital and Research Center, Riyadh, Saudi Arabia; ^5^Western Australian Register of Developmental Anomalies, and Genetic Services of Western Australia, King Edward Memorial, Subiaco, WA, Australia; ^6^Telethon Kids Institute and School of Paediatrics and Child Health, University of Western Australia, Perth, WA, Australia; ^7^Spatial Sciences, Science and Engineering, Curtin University, Perth, WA, Australia; ^8^Department of Psychiatry & Behavioral Science, University of Washington School of Medicine, Seattle, WA, United States; ^9^Alberta Children’s Hospital Research Institute, Calgary, AB, Canada; ^10^National Organization for Rare Disorders, Danbury, CT, United States; ^11^Department of Computer Science, University of Toronto and the Hospital for Sick Children, Toronto, Canada; ^12^Department of Human Genetics, Radboud University Medical Center, Nijmegen, Netherlands; ^13^Manchester Centre for Genomic Medicine, Central Manchester University Hospitals NHS Foundation Trust, MAHSC, Saint Mary’s Hospital, Manchester, United Kingdom; ^14^CHU Nantes, Service de Génétique Médicale, Nantes, France; ^15^Office of Population Health Genomics, Public and Aboriginal Health Division, Department of Health Government of Western Australia, Perth, WA, Australia; ^16^Sir Walter Murdoch School of Policy and International Affairs, Murdoch University; ^17^Centre for Population Health Research, Curtin University of Technology, Perth, WA, Australia; ^18^Hunter Genetics, Waratah, NSW, Australia; ^19^Department of Genome Science, University of Washington School of Medicine, Seattle, WA, United States; ^20^Howard Hughes Medical Institute, University of Washington, Seattle, WA, United States; ^21^Division of Human Genetics, Level 3, Wernher and Beit North, Institute of Infectious Disease and Molecular Medicine, Faculty of Health Sciences, University of Cape Town, Observatory, South Africa; ^22^Wellcome Trust Sanger Institute, Hinxton, Cambridge, United Kingdom; ^23^MRC Human Genetics Unit, IGMM, University of Edinburgh, Western General Hospital, Edinburgh, United Kingdom; ^24^Genetic Services of Western Australia, King Edward Memorial Hospital, Subiaco, WA, Australia; ^25^The Garvan Institute, Sydney, NSW, Australia; ^26^Oregon Health & Science University, Portland, OR, United States; ^27^Wellcome Centre for Ethics and Humanities, University of Oxford, Oxford, United Kingdom; ^28^Ethox Centre, Nuffield Department of Population Health, University of Oxford, Oxford, United Kingdom; ^29^McKusick-Nathans Institute of Genetic Medicine, Johns Hopkins University, Baltimore, MD, United States; ^30^Princess Máxima Center for Pediatric Oncology, Utrecht, Netherlands; ^31^Department of Congenital Heart Disease and Pediatric Cardiology, University Hospital of Schleswig-Holstein–Campus Kiel, Kiel, Germany; ^32^Department of Clinical Neurosciences, Western General Hospital, Edinburgh, United Kingdom; ^33^Oxford Centre for Genomic Medicine, Oxford, United Kingdom; ^34^Department of Medical Genetics, University of Antwerp, Antwerp, Belgium; ^35^Institut für Genomische Statistik und Bioinformatik, Universitätsklinikum Bonn, Rheinische-Friedrich-Wilhelms-Universität, Bonn, Germany; ^36^Great Ormond Street Hospital for Children NHS Foundation Trust, London, United Kingdom; ^37^George A. Jervis Clinic and Institute for Basic Research in Developmental Disabilities (IBR), Staten Island, NY, United States; ^38^Imagine Institute, Paris, France; ^39^Department of Biology and Medical Genetics, 2nd Faculty of Medicine, Charles University and University Hospital, Prague, Czechia; ^40^Laboratorio Chamoles, Errores Congénitos del Metabolismo, Buenos Aires, Argentina; ^41^Department of Paediatrics and Neonates, Fiona Stanley Hospital, Perth, WA, Australia; ^42^CIMR (Wellcome Trust/MRC Building), Cambridge, United Kingdom; ^43^Department of Clinical Genetics, Maastricht University Medical Center, Maastricht, Netherlands; ^44^The Jackson Laboratory, Farmington, CT, United States; ^45^Oasi Research Institute-IRCCS, Troina, Italy; ^46^Victorian Clinical Genetics Service and Murdoch Childrens Research Institute, The Royal Children’s Hospital, Parkville, VIC, Australia; ^47^Northern & Yorkshire Cleft Lip and Palate Service, Royal Victoria Infirmary, Newcastle upon Tyne, United Kingdom; ^48^Department of Molecular and Human Genetics, Baylor College of Medicine, Houston, TX, United States; ^49^Department of Medical Genetics, University and University Hospital Antwerp, Antwerp, Belgium; ^50^Center for Human Genetics, University Hospitals Leuven, University of Leuven, Leuven, Belgium; ^51^Clinical Genetics Group, MRC Weatherall Institute of Molecular Medicine, University of Oxford, John Radcliffe Hospital, Headington, Oxford, United Kingdom

**Keywords:** data sharing, phenotyping, patient information, data protection, rare disease, Faces

## Abstract

The clinical utility of computational phenotyping for both genetic and rare diseases is increasingly appreciated; however, its true potential is yet to be fully realized. Alongside the growing clinical and research availability of sequencing technologies, precise deep and scalable phenotyping is required to serve unmet need in genetic and rare diseases. To improve the lives of individuals affected with rare diseases through deep phenotyping, global big data interrogation is necessary to aid our understanding of disease biology, assist diagnosis, and develop targeted treatment strategies. This includes the application of cutting-edge machine learning methods to image data. As with most digital tools employed in health care, there are ethical and data governance challenges associated with using identifiable personal image data. There are also risks with failing to deliver on the patient benefits of these new technologies, the biggest of which is posed by data siloing. The Minerva Initiative has been designed to enable the public good of deep phenotyping while mitigating these ethical risks. Its open structure, enabling collaboration and data sharing between individuals, clinicians, researchers and private enterprise, is key for delivering precision public health.

## Introduction

All areas of health care stand to benefit from data sharing, but no group perhaps more so than patients with rare disorders affecting facial morphology and their families (Boycott et al., 2017). This paper presents the Minerva Initiative, a global initiative to enable integration of facial photographs and medical information across health care systems and for research. The purpose of this initiative is to empower global research into rare diseases through computational phenotyping from personally identifiable data.

New machine learning approaches hold great promise for transforming health care. By interrogating vast amounts of rich data, they can develop tools that empower clinical care. Making personal data available to researchers across the world raises a number of ethical, legal, data security, and societal challenges. Among the issues one must consider are the following: What is the scope for anonymization of data? How can the rights of individuals be protected in a rapidly changing digital world? How does one enable the potential positive benefits of data sharing? These require the development of new ways of working with, and securely sharing, identifiable data in a scalable and rigorous manner.

Below, we outline the Minerva Initiative—a research data resource (Minerva Image Resource—MIR) and an open research consortium (Minerva Consortium—MC), which has been set up to allow the sharing of identifiable patient data, such as facial photographs and collaborative research projects on rare diseases.

## Improving Rare Disease Diagnostics With Deep Phenotyping

With the advances in clinical and research availability of next-generation sequencing technologies in settings exemplified by initiatives such as the 100,000 genomes project (Caulfield et al., 2017), the All of Us initiatives (“National Institutes of Health (NIH)—All of Us”, n.d.) and the Undiagnosed Diseases Network International (Taruscio et al., 2015; “UDNI”, n.d.), one could be forgiven for thinking that the problem of rare diseases has been largely solved. However, sequencing in clinical settings only aids diagnosis of about 50–60% of rare diseases (Boycott et al., 2017) (selection of patient population influences what this number means in practice), leaving a large group of undiagnosed patients.

We all carry *de novo* and rare variants predicted to have gene-damaging effects that could lead to the variant being interpreted as putatively pathogenic (MacArthur and Tyler-Smith, 2010). Coming to the conclusion that a particular variant is disease contributing for a set of phenotypes is not trivial. There are a number of initiatives to collect information on patient phenotypes, gene, and variants in databases, such as DECIPHER (Wright et al., 2018), PhenomeCentral (Buske et al., 2015a), PhenoDB (Hamosh et al., 2013), GeneMatcher (Sobreira et al., 2015), IRUD (Adachi et al., 2017), KCCG Patient Archive (“Patient Archive”, n.d.), MyGene2 (“MyGene2—Home” 2, n.d.), Human Disease Gene Website series (“Human Disease Genes”, n.d.), ClinVar (Landrum et al., 2016), and the Centers for Mendelian Genomics (Chong et al., 2015). Many of these resources are connected as the Matchmaker Exchange (Philippakis et al., 2015), *via* a common Application Program Interface (Buske et al., 2015b), allowing researchers and clinicians to identify additional patients for novel disorders, and in some cases collect additional cases for known ones. However, as these resources grow, it is no longer safe to infer pathogenic causality just because a putative variant has been observed in another patient previously. As the number of variants of uncertain significance grows, it is expected that the rate of false-positive mutation matches will also increase (Dorschner et al., 2013; Akle et al., 2015; Krawitz et al., 2015). Consequently, the current approach to search disease databases for a mutation match might be insufficient to identify new pathogenic mutations. Patients with very different rare diseases are still likely to have rare mutation matches by random chance. Employing phenotype metrics could add additional power to DNA variant pathogenicity classifications and should feature in efforts to understand genomic variants in clinical settings, such as ClinGen (Rehm et al., 2015). Deep and objective phenotyping delivers an independent source of information that can contribute to inferences about disease-contributing associations (Bone et al., 2016; Pena et al., 2018; Köhler et al., 2017; Robinson et al., 2015). If two patients share a variant of uncertain significance (VUS) and also have similar rare phenotypic manifestations, then the likelihood of the variant being disease contributing is much higher. Certainly, if the putative variants and the combination of clinical metrics are both rare, then the chance of randomly encountering the same combination in an unrelated individual is even less likely (Sifrim et al., 2013; Javed et al., 2014; Singleton et al., 2014; Robinson et al., 2014; Pengelly et al., 2017). Even so, such hypothetical associations should be contextualized in terms of null expectations given variant mutation and population allele frequencies, and additionally verified with functional genetic studies (Akle et al., 2015; Krawitz et al., 2015).

Precise deep phenotyping of patient traits, including facial characteristics, is one such way of aiding the mapping and matching of disease-associated traits. This has the potential to aid the clinical pathways to diagnosis and to empower inference of disease-contributing associations to genetic variants. This promise is contingent on having enough high-quality and accurate data to build methods for extracting disease relevant phenotypes, but also the numbers to link rare and ultra-rare disorders. Translating deep phenotyping approaches to clinical utility is a big data challenge.

## Phenotyping From Photographs

Developments in computer vision and deep learning are being applied to patient datasets with the aim of aiding diagnosis, prediction of outcomes, and monitoring of clinical phenotypes. One of the first clinical settings where this is being applied is clinical dysmorphology for rare diseases.

It should be noted that the idea to objectively assess body form (anthropometrics) in Western medicine was pioneered by Francis Galton well over a century ago (Galton, 1879) and applied to images by Sheldon et al., in 1940 (Sheldon et al., 1945). However, the formalized discipline of clinical dysmorphology, as the study of birth defects, was not conceptualized until 1966 (Smith, 1966). The challenge of bringing to bear machine learning and data analytic approaches to clinical dysmorphology, and anthropometrics from image data, has been the target of many threads of research. Various research endeavors for extracting objective phenotype metrics have been applied to both 2D (Herpers et al., 1993; Loos et al., 2003; Vollmar et al., 2008; Balliu et al., 2014) and 3D craniofacial imaging (Hammond et al., 2004; Hammond et al., 2005; Hennessy et al., 2010; Hammond et al., 2012; Baynam G. et al., 2013; Baynam G.S. et al., 2013; Kung et al., 2015; Baynam et al., 2016).

Despite the precision and objectivity of these methods, their practical application has been relatively limited. This can be largely attributed to a combination of expensive instrumentation, a requirement that patients are able to pose for imaging, and the need for expertise for the manual steps in subsequent data analyses. Recent developments in imaging platforms and machine learning capabilities provide the foundation for applications with greater clinical utility. High-quality 2D digital imaging cameras have become ubiquitously available, and 3D capabilities are beginning to enter the consumer market. Also, cutting-edge deep convolutional neural networks approaches are transforming the way these images can be analyzed. It has been shown that they can be trained to be highly robust for imaging variation, reducing the need for highly controlled subject poses (Xiangyu Zhu et al., 2015). There are a number of current research and commercial efforts to create fully automated analysis pipelines for clinical interpretation of dysmorphologies (Ansari et al., 2014; Ferry et al., 2014; Manousaki et al., 2015; Basel-Vanagaite et al., 2016; Gripp et al., 2016; Baynam et al., 2017; Bengani et al., 2017; Dudding-Byth et al., 2017; Deciphering Developmental Disorders Study, 2017; Gardner et al., 2017; Hadj-Rabia et al., 2017; Kruszka et al., 2017a; Kruszka et al., 2017b; Kruszka et al., 2017c; Lumaka et al., 2017; Shukla et al., 2017; Valentine et al., 2017; Reijnders et al., 2018b; Gurovich et al., 2018; Knaus et al., 2018; Kruszka et al., 2018; Liehr et al., 2018; Pantel et al., 2018; Reijnders et al., 2018a; Reijnders et al., 2018b; Zarate et al., 2018). However, all these efforts are meeting the same barriers to progression of the methods and prospects for clinical impact, challenges to do with data access, ethics, governance, and security. Without addressing these issues on a common basis, these efforts will struggle to deliver on their potential patient benefits.

## Big Data Challenges

There is a long history of the use of medical algorithms. The clinical process of establishing a diagnosis and suitable treatment is essentially a step-wise application of decisions facilitated by objective tools and metrics and augmented by cumulative knowledge and experience. Artificial intelligence, or machine learning, in health care employs the same principles to improve and accelerate clinical pathways. New machine learning approaches could add value in health care sectors challenged by extreme data volumes or complexity of inferences needed.

While there is a noticeable rush of “big data” applications in health care, the delivered clinical utility has so far been very limited. This lack of clinical utility has been attributed to the poor model interpretability or performance on real world data. All deep learning approaches are reliant on large datasets to learn from in order to perform accurately. Identifiable medical datasets are typically relatively small in the context of deep learning, and data are usually sourced from single health care settings or specific populations. This makes models over fit, that is to say, become overly specific to the particular datasets they have been shown, and consequently, they perform poorly on new datasets. To efficiently use big data approaches, we need to address the challenges of working with data outside of the isolated silos of single projects or health care systems.

Ethical, legal, data security, and intellectual property issues are key barriers preventing the ascertainment, interrogation of big datasets, and the implementation of big data solutions in health care. Traditional research consent structures may constrain or prevent data sharing and the integration of datasets essential for these new approaches. In the context of the strict regulation of and accountability in health care data, if there are any doubts about the rules around sharing, the default position is to not share. Consequently, data sharing is unlikely to happen without specific consent for integration of personally identifiable information. Consequently, most data-sharing efforts bringing together global data rely heavily on de-identification or the minimization of sharing more sensitive data (Dyke et al., 2017).

The reality of big data is that anonymization might not be possible (Gymrek et al., 2013; de Montjoye et al., 2015). Furthermore, data security concerns for data access rights and the prospects of malicious digital activity (hacking) imply often unacceptable risk where organizations are liable for data loss. Finally, data sharing is inhibited by concerns about intellectual property rights: who owns the data and to whom do any findings or inventions derived from the data belong? In practice, these factors all tend to result in data siloing, since the easiest way to control the use of data is to keep it hidden. However, such practices hamper the prospects of new public good, increased clinical utility, and impact.

Moreover, data silos inevitably build in data biases, which create implicitly biased models, which in turn limit generalization to new data. For big medical data to deliver on its potential clinical utility, generally and equitably, approaches must be able to generalize across health care systems and different populations. Such an approach will deliver solutions from a basis of “n = 1” approach prevalent in precision medicine approaches to an “n = many” impact under a precision public health paradigm (Collins and Varmus, 2015; Weeramanthri et al., 2018; “Precision Public Health: What Is It? | | Blogs | CDC”, n.d.).

## Minerva Initiative

The Minerva Initiative has been developed to enable research into the use of image analysis for the diagnosis of diseases, prevent data siloing, and foster further healthy competition between various image phenotyping approaches. The Minerva Initiative is an effort to construct a precompetitive space for enabling research on clinical phenotyping tool development. It has been constructed in the spirit of open science (Woelfle et al., 2011; Nielsen, 2012) and within the bounds of ethics and data governance constraints.

The Minerva Initiative has the following objectives: to build a community of researchers and clinicians; to continue to develop ethical structures and provisions for working on identifiable clinical images; and to deliver secure data sharing between consortium members. It has been constructed to align with the goals and objectives of the Global Alliance for Genomics & Health (“Global Alliance for Genomics and Health”, n.d.) and the International Rare Diseases Research Consortium (IRDiRC) (“IRDiRC”, n.d.).

## Minerva Consortium

The Minerva Consortium (MC) is an international network of clinicians and researchers, from both public and private organizations, involved in the Minerva Initiative. Within the MC, there are many purposes and interests, but all MC members seek to establish a commonly agreed set of conduct and praxis for the benefit of all stakeholders: patients, participants, researchers, clinicians, and social systems.

The MC consists of a management group, clinical collaborators, working groups, and phenotyping groups. The management group is tasked with guiding directions of joint MC efforts, MC policy and strategy, acceptance of new MC Computational Phenotyping Groups, and dispute resolution. For prospective data collected under the Minerva Initiative consent, the Management Group also acts as a Data Access Committee overseeing Phenotyping Groups’ access to the Minerva Image Resource. The goal of the MC is not to consolidate all phenotyping projects to one unified approach but rather to foster an environment to enable a multiplicity of methods to develop.

## Minerva Image Resource

To improve the models for phenotype descriptions through deep learning, data need to be brought together in a unified compute system. Distributed systems are not feasible because either they sacrifice security, by virtue of exchange of semi-identifiable data, or because data transfer time and volumes are increased to such a level they are not workable. While fixed algorithms can be deployed in distributed frameworks, this would also prevent iterative learning regimens, which are required for continuing improvements. Consequently, the Minerva Consortium has focused on legal, ethical, and data governance structures to allow the global pooling of data in a unified system.

The Minerva Image Resource (MIR) is a centralized repository of personally identifiable data, which covers both images and linked medical data. While the facial images are inherently identifiable, other directly identifiable data, such as names, addresses, social security numbers, or any direct hospital identifiers, are not stored in the MIR. Coded identifiers, supporting privacy preserving record linkage, are stored in the MIR, but the linkage to any other data remains with the original data controllers—but are expected to be available through Matchmaker Exchange linked initiatives (Philippakis et al., 2015).

## Ethics and Data Flow

### Retrospective Data

Patient datasets that comprise collected data and images from previous projects can be joined into the MIR if the existing consent allows for data use for general research purposes. The threshold that should be met for data to be contributed to the MIR is consented for use in research, not necessarily consented for publication. No identifiable information held in the MIR is shared outside the scope of legal agreements covering agreed research purposes.

The MC has a defined standardized Material Transfer Agreement and collaborator letter that is recommended for use within the consortium, but ultimately it is up to each clinical collaborator and phenotyping group to sign any given agreement. It is up to MC Clinical Collaborators to vouch that this consent is in place and that the wording of the patient consent encompasses image data. This is also specified in the Terms and Conditions for the Material Transfer Agreements. The expectation is that data should be shared as openly as possible within the consortium within the permissible scope of consent and data governance restrictions.

### Prospective Data

Clinicians can recruit patients into the Minerva Initiative if they obtain consent for the use of patient’s data and images collected through routine clinical practice. The Minerva Consortium have drafted patient information and consent forms, but locally valid research ethics approvals need to be sought for the inclusion of patient data and images in Minerva Consortium research. The patient consent forms cover a broad remit of health-related research using images and associated clinical data.

Once research ethics committee-approved based consent has been given and a Data Submission Agreement is in place, data can be entered into the MIR and become part of the Minerva Initiative. The data flow pathway for this is not prescriptive. The data could, in future, be deposited in the MIR directly or through affiliated third-party apps or other health care record platforms.

Data collected prospectively through clinical collaborators using the Minerva Initiative consent will be shared between all Phenotyping Groups in good standing with the Minerva Consortium (having been approved by the MC Management Group and having a legal agreement for data access with the MIR). To be clear, in line with these structures, the original photographs will thereby be shared with these phenotyping groups.

### Public Participation

A public website has been constructed, Minerva&Me (https:///www.minervaandme.com) (“Minerva and Me—Help Rare Disease Research”, n.d.), which allows anyone around the world to participate directly in the Minerva Initiative. Through Minerva&Me participants are able to enter basic information about themselves and any medical diagnoses they might have and to upload images. Photographs of themselves can be “selfies” or scanned images from photographs in existing family albums. Participants retain control of the use of their image and associated personal data. Minerva&Me employs a dynamic consent model (Kaye et al., 2015; “Platform for Engaging Everyone Responsibly | GeneticAlliance.Org”, n.d.), whereby participants are able to amend their consent or delete their data from the MIR directly.

Minerva&Me has been reviewed and approved by a research ethics committee (Oxford Tropical Research Ethics Committee at the University of Oxford), and also has a governing Advisory Board. This Advisory Board has representatives from clinicians, lawyers, data security experts, and patient advocacy groups including Unique, NORD, Rare Voices Australia, and Rare Disease UK. This Advisory Board has oversight of any future developments and directions for Minerva&Me.

In future versions of Minerva&Me, we intend to allow participants the option to enable their doctor to have limited access to their data analyses through coded linkers. The purpose of such links would be to allow further clinical information to be integrated by the MC Clinical Collaborator and, in future, allow a potential feedback route through the clinician to patient (if and when returnable findings are generated). Consent sought between patient and clinician must then cover the use of clinical data through the Minerva Consortium and the linkage to Minerva&Me through coded identifiers.

### Analyzed Data

Identifiable data within the MIR is only available to the original data submitters or MC Computational Phenotyping Groups. MC Computational Phenotyping Group researchers could be from all over the world and may be working in academia or commercial companies. Access to the Minerva Image Resource is only granted for purposes that align with the research goals of the Minerva Initiative (to improve diagnosis, clinical treatment, and understanding of a wide range of illnesses). Data access conditions are further explained below.

**Figure 1:**
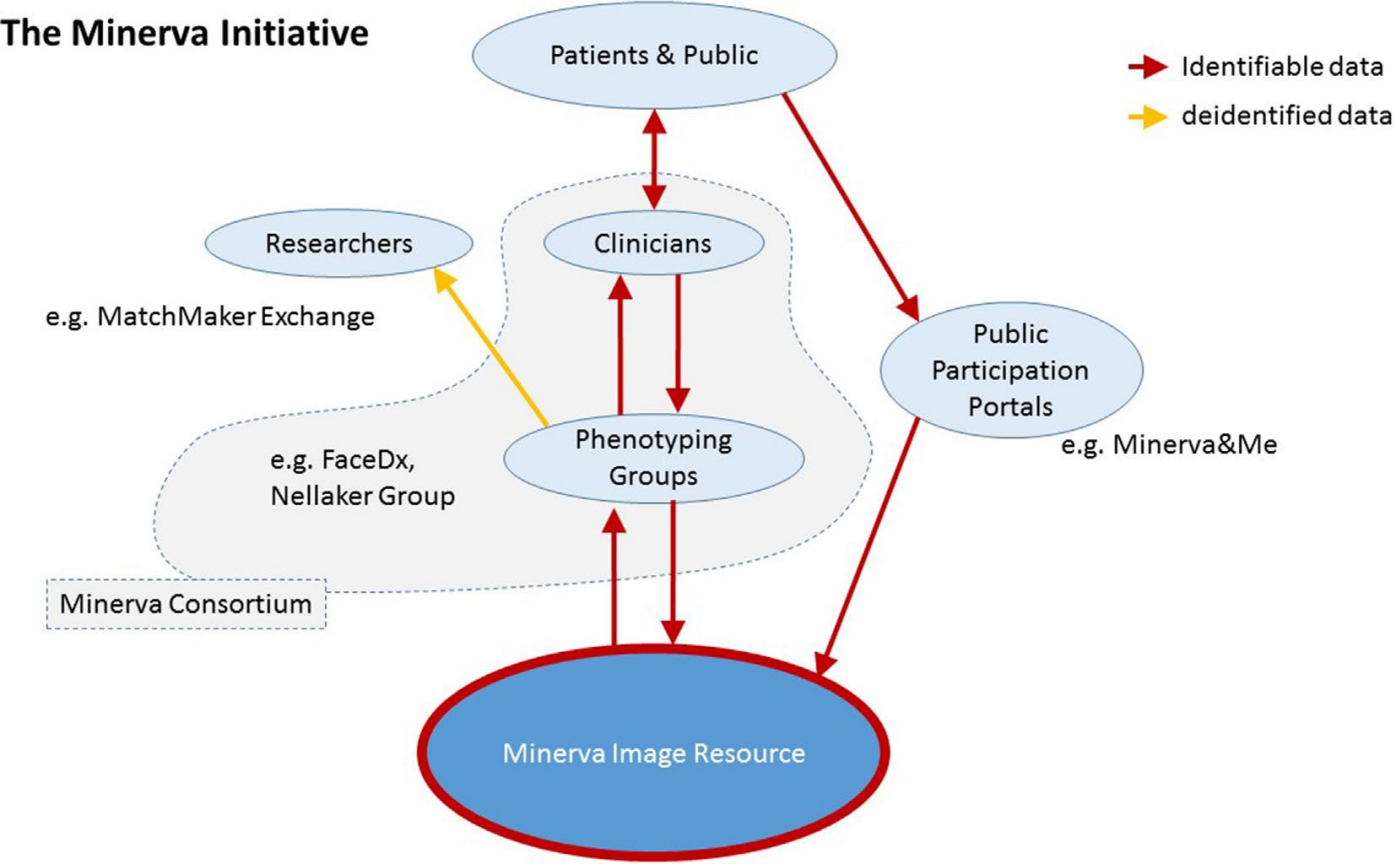
Schematic overview of the Minerva Initiative structure and data flow into the Minerva Image Resource.

The MIR, as a precompetitive space, does not enforce a single approach or initiative but rather seeks to encourage multiple efforts. There is the expectation that results from analyses on the images and identifiable data will be returned and shared within the MC. This is to enable comparisons between different approaches using commonly agreed upon testing sets but also to keep the science as open and collaborative as possible. A schematic overview of the flow of data in the Minerva Initiative is shown in **Figure 1**.

## Collaboration Enabling Network

One of the purposes of the MC is to encourage and enable collaborative work on identifiable patient data. These collaborative initiatives are not centrally coordinated but are expected to be conducted through normal scientific practice. In addition, there is a framework for collective attribution through a publication policy.

Any data submitted that contribute to a publication using the pooled dataset will be acknowledged in accordance with the guidelines of the International Committee of Medical Journal Editors (“ICMJE | Recommendations | Defining the Role of Authors and Contributors”, n.d.). We envisage two broad groups of publications to arise from project data, each with distinct guidelines for recognizing contributions:

- “Core” MC papers have a broad focus and use large amounts of data from multiple aspects of the project. Named authors (details below) plus “Minerva Consortium” with all project participants listed in the end matter [cf. Mells et al. WTCCC3 paper (Mells et al., 2011)]
- “Affiliated” MC papers arising from more specific collaborations focused on a particular phenotype, methodology, policy question, etc. Named authors based on contribution and the Minerva Consortium as a “corporate” author [project participants not listed, cf. Firth, Wright & DDD Study paper (Firth et al., 2011)].

Manuscripts derived from research conducted on data acquired through the Minerva Initiative are reviewed by a Publication Review Group to affirm that author attribution follows the publication policy. The publication review group also ensures that all statements, and images used in Minerva Initiative publications comply with participant consents, image usage rights, data privacy, and data governance considerations.

## Identifiable Information Flow and Intellectual Property

While analyses produced by models are shared within the Minerva Initiative, the initiative does not require that the models themselves are shared. This is to ensure data protection and intellectual property rights. For many models being trained, there is a large gap in understanding of interpretability—to the degree that there is no guarantee that personally identifiable information from individuals in the training data might not be recoverable. Thus, to assure future compliance with the protection of individuals’ data rights within the MIR, the models must not be shared without corresponding data governance assurances. Secondly, as the Minerva Initiative allows the coexistence of both academic and commercial enterprise initiatives, the fair and equitable basis of IP domains must be clear.

In other words, in line with personality rights, ownership of the data and photographs remains with the original creators (the person in the image). The means to compare data between people, the models, is where we expect new intellectual property to be created.

It should also be noted that for most anticipated uses, models, in themselves, will contribute very little without access to the datasets. To successfully identify patients with rare disease and match them with others around the world, broad and unfettered interrogation of data is key. No model will have the accuracy or confidence to make assertions about rare diseases without reference back to the original patients and data. Consequently, it is in *every* stakeholder’s interest that the data are shared as openly and as widely as possible.

## Joining the Minerva Consortium and Data Access Conditions

As a clinician or research academic seeking to collaborate on de-identified data or wishing to submit consented patient data to the MIR for analysis, the Minerva Initiative is open to join. The criteria are a formal affiliation and good standing with professional organizations in either a clinical or academic capacity. The Minerva Consortium relies on a peer system of oversight on membership, with the Management Group having power to rule in disputes.

In the case of a group seeking to become a Phenotyping Group, who can access identifiable data held in the MIR, this status should only be granted to groups who align with the research goals of the Minerva Initiative (to improve diagnosis, clinical treatment, and understanding of a wide range of illnesses). In addition, the Management Group will assess the stated purpose, ethics approvals, data management plans, and legal status of the applicant organization and proposed legal agreement for access to the MIR. Legal agreements will specify the parties’ obligations to adhere to the allowed uses and purposes for the data and the rights to change, revoke, or enforce these conditions (i.e., how access can be revoked and who is liable for possible misuses).

## Conclusion

The Minerva Initiative is a framework for global collaboration on identifiable patient data including photographs. It is designed to complement current initiatives for global data sharing in rare diseases by specifically addressing the analysis of data where anonymization is not possible. The Minerva Initiative directly addresses the ethical, legal, and data security challenges associated with inherently identifiable data, thereby enabling big data research. We envisage that the frameworks established within the Minerva Initiative will provide a useful community model to ensure that the amount or variety of identifiable data that any one group, institution, or country can assemble is no longer the limiting factor for advancements in clinical translation of new machine learning methods. Through these approaches, we aim to improve diagnostic rate and classification of DNA variants with a focus on rare diseases. Accordingly, we envisage that this will assist in achieving the International Rare Diseases Consortium Vision, specifically to enable all people living with a rare disease to receive an accurate diagnosis, care, and available therapy within 1 year of coming to medical attention.

## Author Contributions

All authors contributed to the conception and design of the Minerva Initiative. CN drafted the manuscript. All authors contributed to manuscript revision and read and approved the submitted version. 

## Funding

This work was supported by MRC Fellowship MR/M014568/1 to CN and MRC Grant MR/M01326X/1 for MF. RFK and AVD are supported by grants from the ERA-NET NEURON through the Research Foundation – Flanders (FWO). EEE is supported by NIH 5R01MH101221 and as an investigator of Howard Hughes Medical Institute. CR acknowledges funding by the Italian Ministry of Health, Project RC2019 No. 2751604.

## Conflict of Interest Statement

The authors declare that the research was conducted in the absence of any commercial or financial relationships that could be construed as a potential conflict of interest.
